# Supplementary figures and images for: Rare ground data confirm significant warming and drying in western equatorial Africa
Source: PeerJ. 2020 Apr 14;8:e8732. doi: 10.7717/peerj.8732 (PMC7164428; doi:10.7717/peerj.8732)

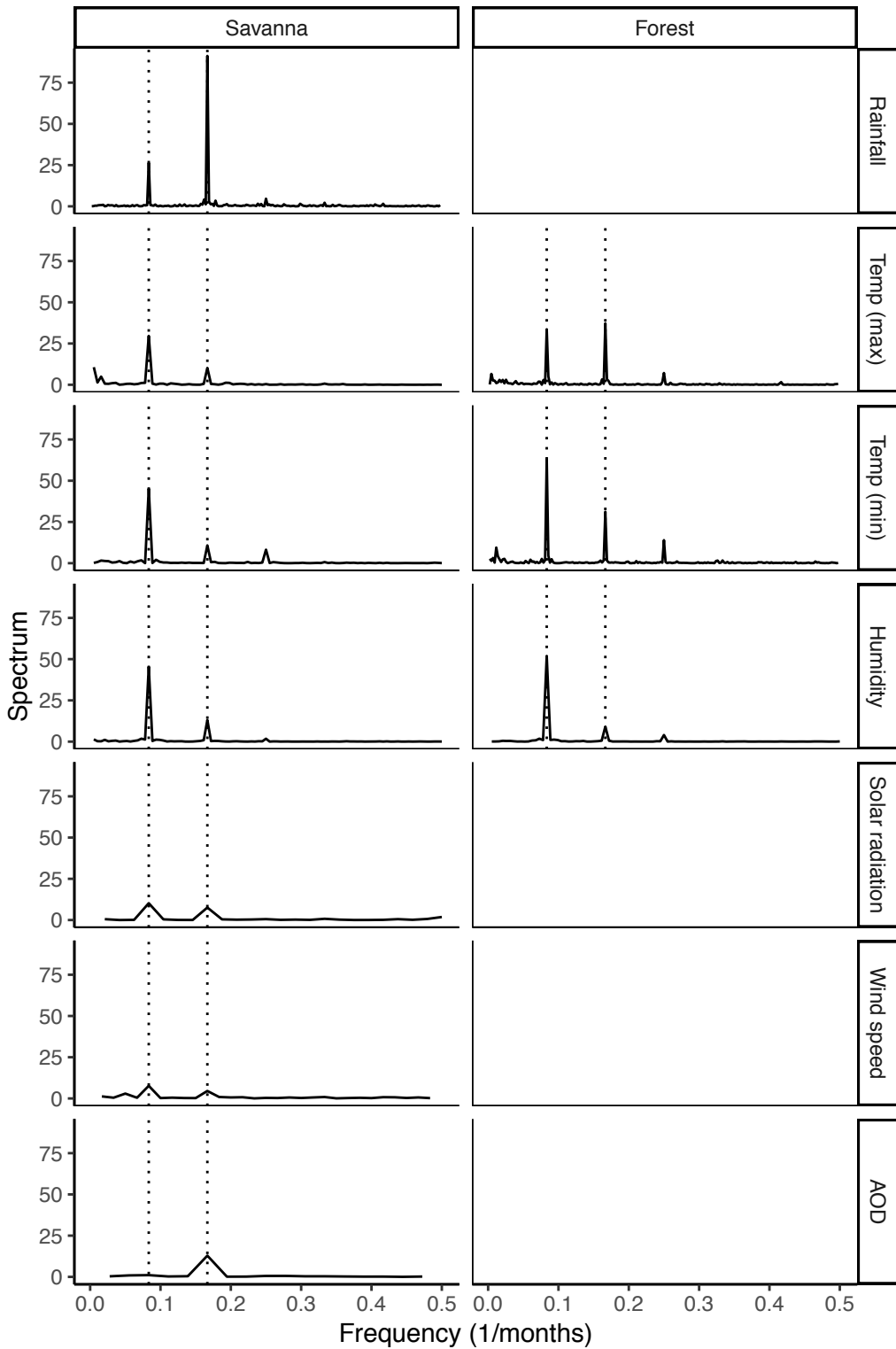

Supplement: Supplemental Information 1 — Fourier spectra represent the power of the cycle at each possible frequency. Peaks in the spectra indicate dominant cycles in the data. The dotted vertical lines indicate the position of annual (1/12 = 0.083 cycles per month) and biannual (1/6 = 0.167) cycles. Time series were standardised but observation lengths differ. [file peerj-08-8732-s001.pdf]

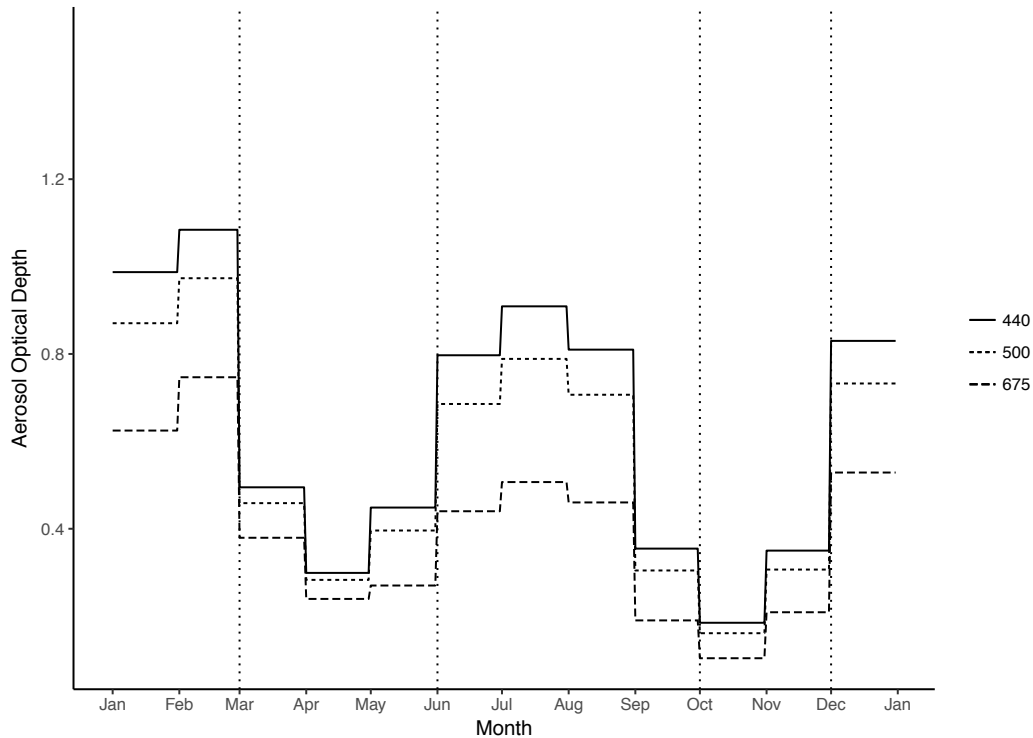

Supplement: Supplemental Information 2 — Lines indicate the monthly means calculated from daily data. The three wavelengths (440 nm, 500 nm and 675 nm) are all relevant for photosynthetically active radiation. [file peerj-08-8732-s002.pdf]

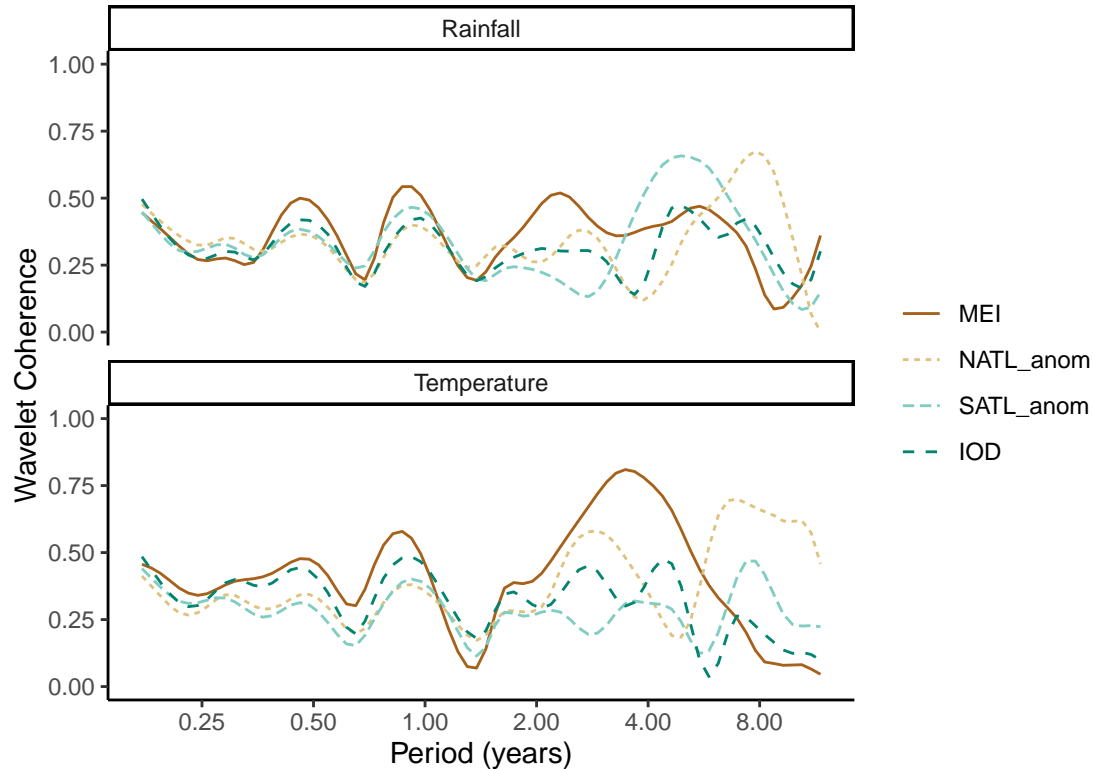

Supplement: Supplemental Information 3 — This plot shows time-averaged wavelet coherence for all eight combinations of x (rainfall or minimum temperature) against y (MEI = Multivariate ENSO Index, NATL = northern tropical Atlantic SST, SATL= southern equatorial Atlantic SST, IOD = Indian Ocean Dipole). Wavelet coherence falling within the cone of influence (where edge effects make the data unreliable) was removed before calculating the mean per period. Wavelet coherence indicates how well the time series are correlated at each period over time and ranges from 0 to 1. Figure S3 shows the individual wavelet coherence plots from which these are derived. [file peerj-08-8732-s003.pdf]
