# Supplementary material for: Rare ground data confirm significant warming and drying in western equatorial Africa
Source: PeerJ. 2020 Apr 14;8:e8732. doi: 10.7717/peerj.8732 (PMC7164428; doi:10.7717/peerj.8732)
Supplement: Supplemental Information 5 [file peerj-08-8732-s005.pdf]

Supplemental Article S1

Lopé weather data selection and preparation

1. Summary of rainfall data recorded at Lopé NP, Gabon (1984 – 2018) and derived datasets 2
1.1 Sources of rainfall data at Lopé NP 2
1. 2. Data standardisation and selection 2
2. Summary of temperature data recorded at Lopé NP Gabon (1984-2018) and derived datasets 6
2.1 Sources of temperature data at Lopé NP 6
2.2. Addressing major challenges and data selection 7
3. Summary of humidity data recorded at Lopé NP, Gabon (1984 – 2018) and derived datasets 11
3.1 Sources of humidity data 11
3.2. Addressing major challenges and data selection 12
4. Summary of wind speed data recorded at Lopé NP, Gabon (2012 – 2016) and derived datasets 14
4.1 Sources of wind speed data 14
4.1 Data selection 14
5. Summary of solar radiation data recorded at Lopé NP, Gabon (2012 – 2016) and derived datasets 16
5.1 Sources of solar radiation data 16
5.2 Data selection 16
6. Summary of aerosol optical thickness data recorded at Lopé NP, Gabon (2014–2018) and derived datasets 18
6.1 Source of aerosol optical thickness data 18
6.2 Data selection 18

1. Summary of rainfall data recorded at Lopé NP, Gabon (1984 – 2018) and derived datasets

1.1 Sources of rainfall data at Lopé NP

Rainfall has been recorded at Lopé NP using two pieces of equipment: a manual rain gauge (total precipitation for the preceding 24 hour period recorded at 8am each day) and a weather station. The Davis VantagePro2 weather station (https://www.davisinstruments.com/solution/vantage-pro2/) was installed in January 2012 and recorded rainfall every 30 minutes for two years until the equipment was struck by lightning in January 2014. We calculated daily rainfall from the weather station data using a 24-hr period beginning and ending at 8am to match the rain gauge (Figure 1.1).

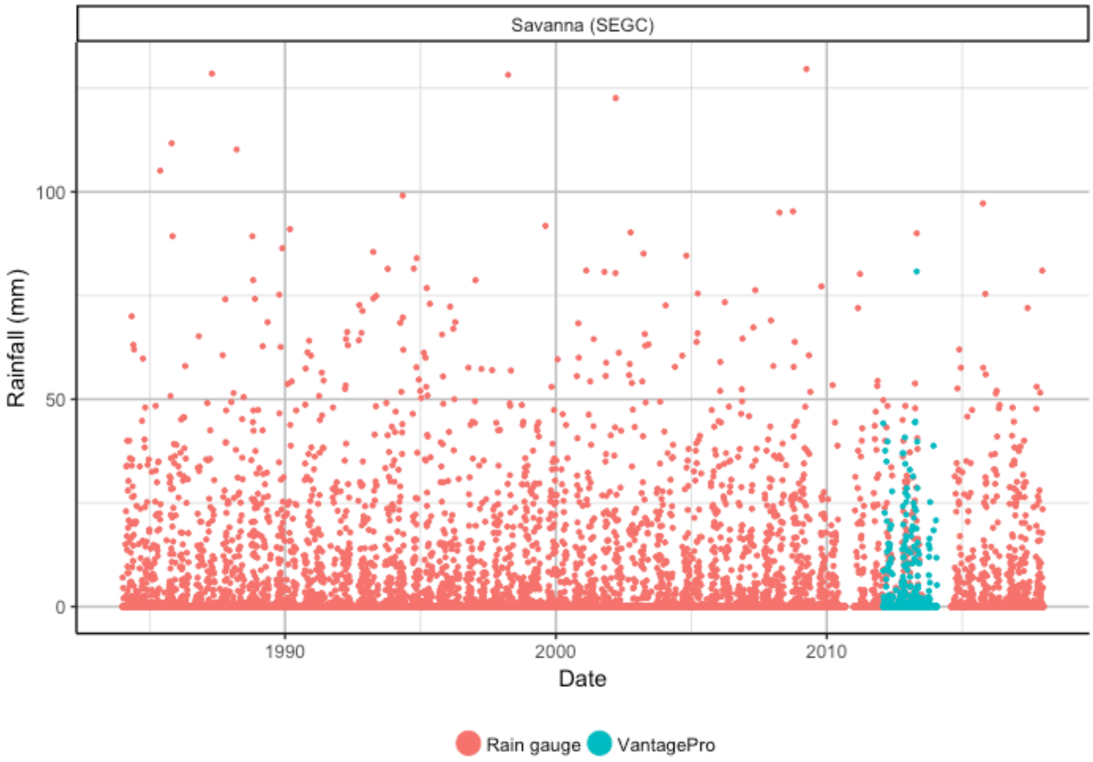

Figure 1.1. Time series plot of rainfall observations at Lopé NP, 1984-2018. *Coloured dots show original daily observations from both the rain gauge and the VantagePro weather station.*

1. 2. Data standardisation and selection

When we compared simultaneous measurements of total daily rainfall from the two different equipment recorded between 2012 and 2014 we found that the weather station consistently underestimated rainfall compared to the rain gauge (Figure 1.2).

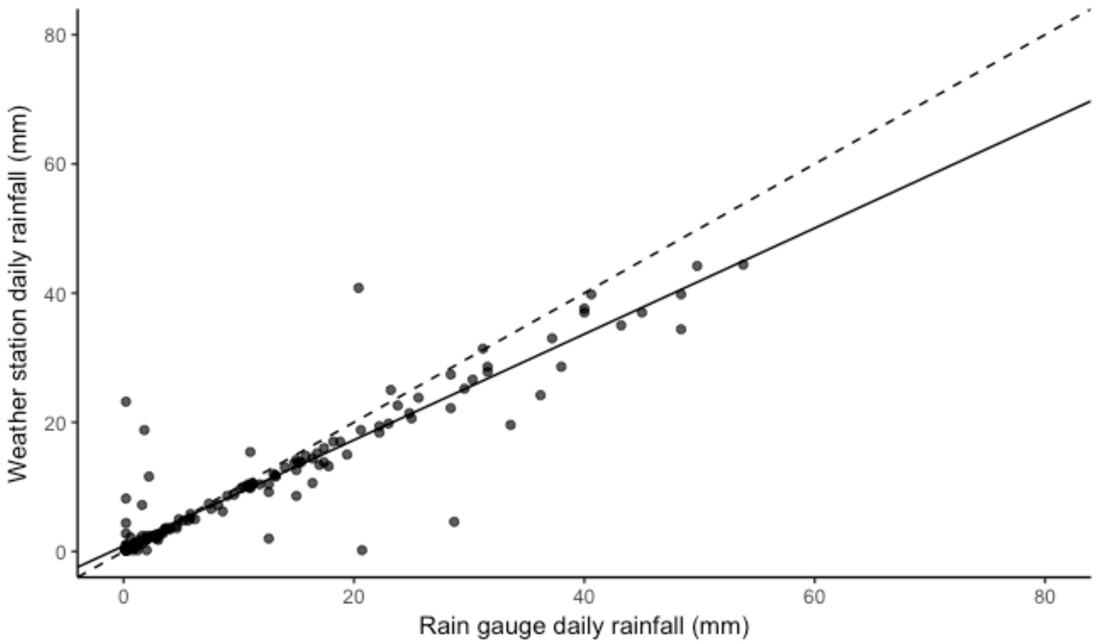

Figure 1.2. Comparison of simultaneous non-zero rainfall observations from the weather station and the rain gauge at Lopé NP, 2012-2014. The dotted line indicates the expected 1:1 relationship. The solid line indicates the model prediction.

In order to standardise the data record we evaluated the relationship between rain gauge-rainfall and weather station-rainfall for all simultaneous non-zero daily records within a linear model (Table 1.1). We extracted the intercept and slope from the linear model and used these values to adjust the weather station data accordingly.

Table 1.1. Estimates from a linear model used to standardise Lopé rainfall observations between equipment. Rainfall observations from the rain gauge were used to predict rainfall observations from the weather station (VantagePro) for all simultaneous non-zero observations.

Predictor	Estimate	SE	T value	P value
Intercept	0.86	0.42	2.05	<0.05
Rainfall (rain gauge)	0.82	0.02	35.67	<0.0001

The dataset available from the University of Stirling's DataSTORRE (http://hdl.handle.net/11667/133) is the standardised daily rainfall data derived from both pieces of equipment (rainfall daily dataset C).

First we calculated mean daily rainfall for each date in the time series with more than one record to create a complete time series for daily rainfall (rainfall daily dataset D). Between 1st January 1984 and 31st December 2017 there were 369 days with no rainfall observations (3% total number of days). Where possible we filled these gaps using the 10-day running mean for the time-series, but 308 missing daily observations remained in three blocks: 2010-09-16 - 2010-12-26 (lost rain gauge data), 2013-10-31 - 2013-11-30 and 2014-02-02 - 2014-07-26 (lost VantagePro data due to lightning strike on equipment). Both non-interpolated and interpolated daily rainfall totals can be found in Rainfall daily dataset D. We created a monthly time series for rainfall by summing

interpolated daily rainfall for each month in the record. Certain analyses require complete time series and so we filled missing months using the mean value for corresponding calendar month (non-interpolated and interpolated monthly data found in rainfall daily dataset E; Figure 1.3).

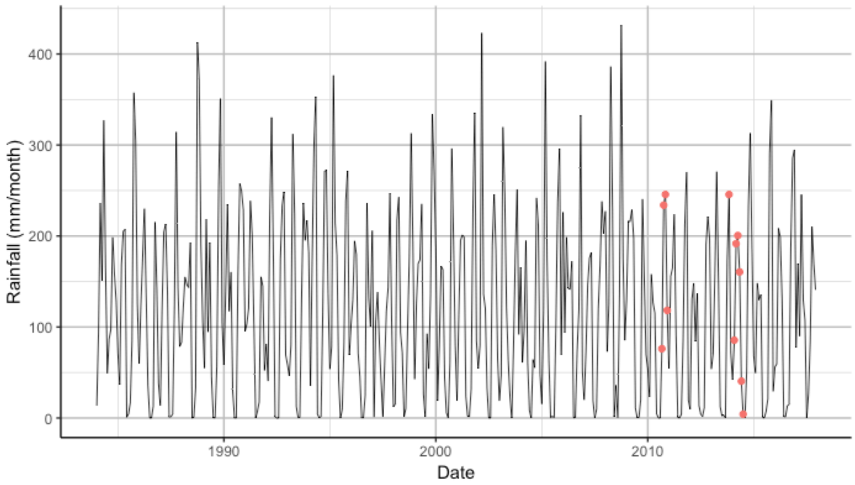

Figure 1.3. Time series plot of monthly rainfall at Lopé NP, 1984-2018 (Dataset E). The line shows the monthly time series. Red dots indicate missing months, which were filled using the mean value for the corresponding calendar month.

Finally, we created an annual time series for rainfall by summing interpolated daily rainfall for each year in the record (Rainfall annual dataset F). Three years (2010, 2013 and 2014) are missing from this annual time series due to lack of data.

All data selection and manipulation procedures described above can be followed in the accompanying flow chart (Figure 1.4) and match the accompanying R code (Code S1).

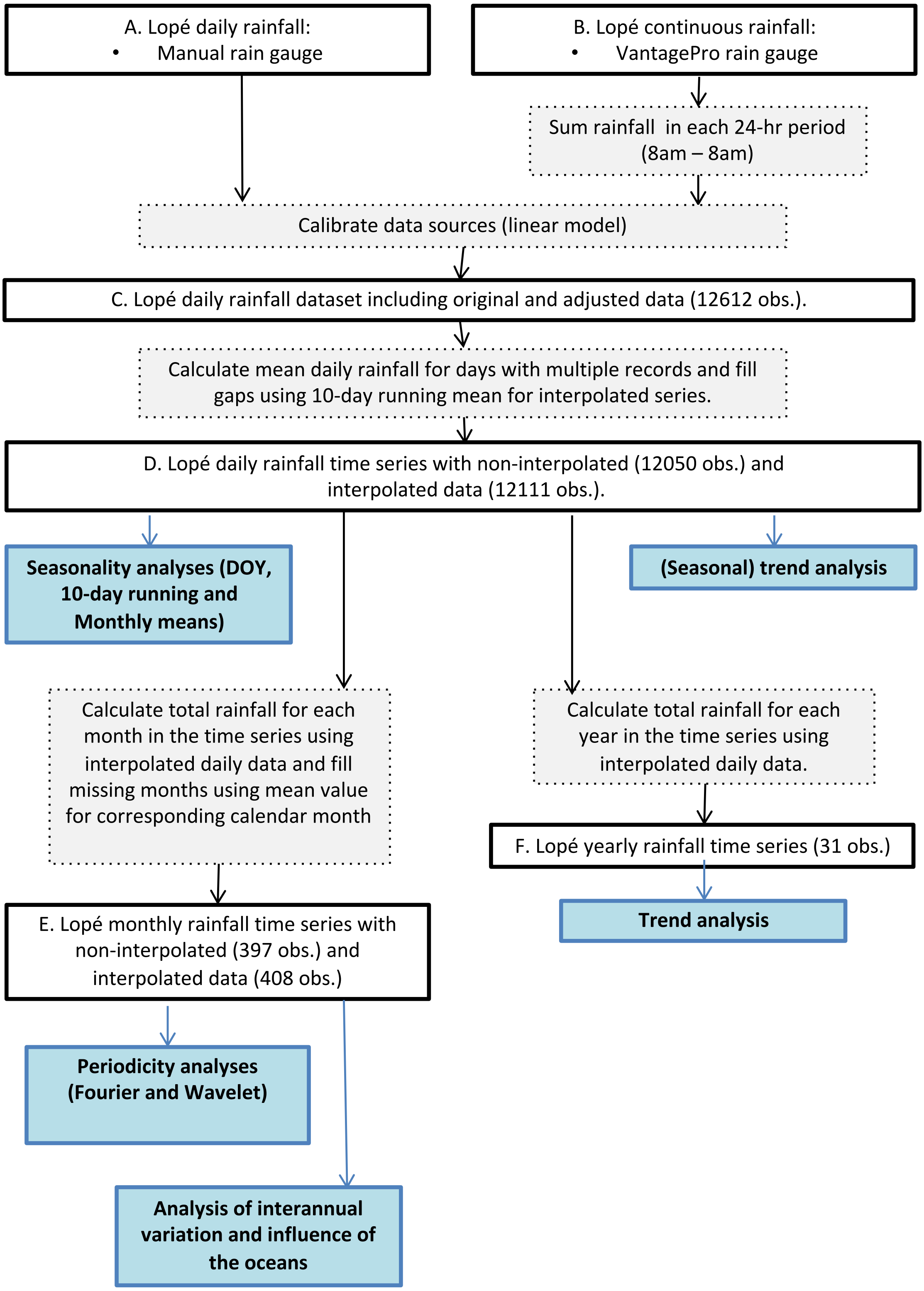

Figure 1.4. Flow chart to show data selection and manipulation steps for Lopé rainfall analyses. Bold boxes indicate derived datasets, grey dashed boxes indicate processes and blue boxes indicate subsequent analyses.

2. Summary of temperature data recorded at Lopé NP Gabon (1984-2018) and derived datasets

2.1 Sources of temperature data at Lopé NP

Maximum and minimum daily temperatures have been recorded at Lopé using six different pieces of equipment at two sites (a savanna site: 11.605E, -0.201N and a forest site: 11.605E, -0.206N) from 1984 to the present.

A manual max/min thermometer was located at the forest site (1.5m aboveground under closed canopy), from 1984 and was checked whenever field teams passed it or daily when logistics permitted. The thermometer recorded the highest and lowest temperature since last reset and data was recorded at irregular intervals. In the case of multi-day intervals between data observations it is impossible to know which date temperature extremes occurred on. We assigned the recorded observations to the mid-date between the current and previous observations for all multiday intervals outside of three major breaks where the equipment was out of use: 1998/07 - 1999/01, 2001/03 - 2001/08 and 2001/08 - 2006/06. In 2002 all temperature recording at the forest site was transferred to continuous automatic units (ONSET HOBO® Data Loggers refhttps://www.onsetcomp.com/, these units also recorded relative humidity). At the same time temperature recording using the same units also began at the savanna site. Due to technical failures these units were replaced in 2006 with the original manual max/min thermometer in the forest and a digital max/min thermometer (Taylor 1441) in the savanna. The digital max/min thermometer detected the highest and lowest temperatures of the previous 24hrs (usually recorded between 8 and 9am). The thermometers were once again replaced by another type of automated unit (TinyTag Plus 2, Gemini Data Loggers https://www.geminidataloggers.com/data-loggers/tinytag-plus-2, some of which recorded both temperature and relative humidity), deployed in the forest from 2007 and in the savanna from 2008 and used until the present (with a gap at the forest site from mid-2015 to mid-2016 and intermittent recording throughout 2017 partly due to termite infestation).

Two weather stations - a Davis VantagePro2 (https://www.davisinstruments.com/solution/vantage-pro2/) and a SKYE MINIMET weather station (https://www.skyeinstruments.com/minimet-automatic-weather-station/) - were installed at the savanna site (sited near the research station, on a rock 4m from the ground) between 2012 and 2016. The VantagePro recorded data every 30 minutes for two years until the equipment was struck by lightning in January 2014. The SKYE station was installed in 2013 and collected data intermittently until 2016 when the equipment was also damaged by lightning: data records between January 2014 and November 2014 were also lost

The automatic units and weather stations collected data in intervals up to 30 minutes long. We summarised these data by calculating the minimum and maximum daily temperature for each 24hr period from 8am-8am to match the thermometer data (Figure 2.1).

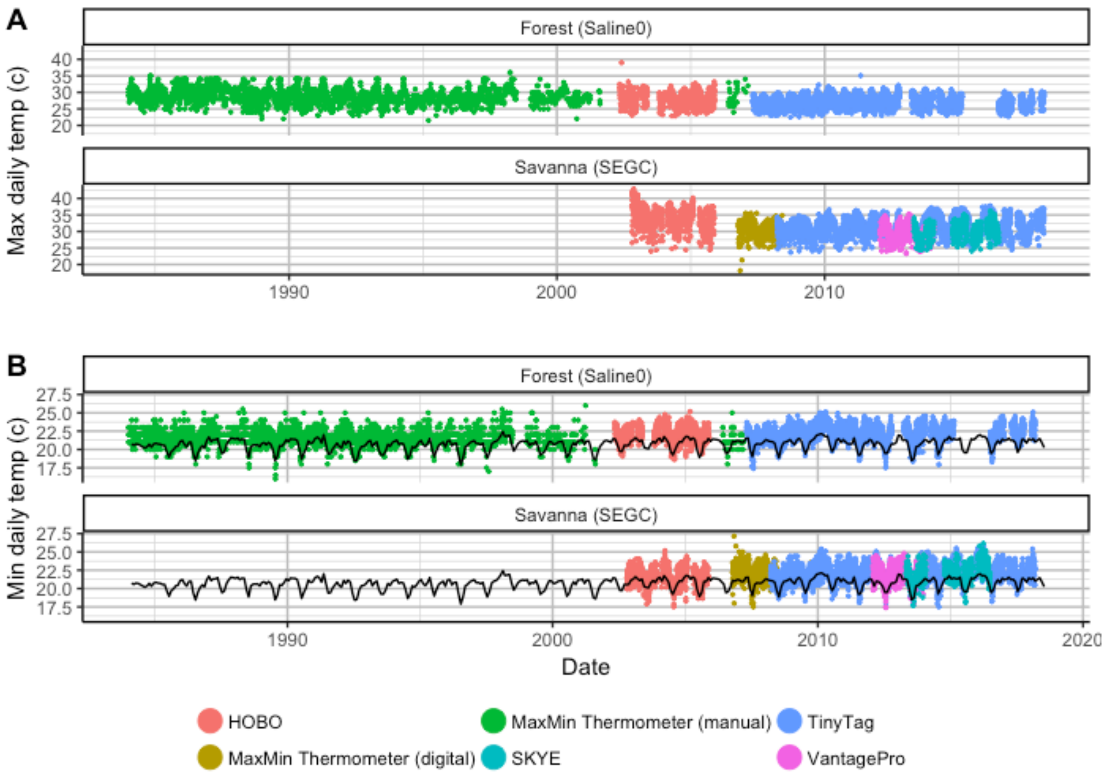

Figure 2.1. Time series plots of maximum (A) and minimum (B) daily temperature observations at Lopé NP, 1984-2018. Coloured dots show the original mean daily observations from different equipment. The black line shows monthly mean minimum daily temperature from the Berkeley dataset.

We combined the continuous data with the manual and digital thermometer data to form the daily maximum and minimum temperature record available for download from the University of Stirling's DataSTORRE (http://hdl.handle.net/11667/133) and is referred to as temperature daily dataset C.

2.2. Addressing major challenges and data selection

The three major challenges in using the Lopé temperature record for long-term analyses are: 1) the impacts of direct solar radiation on equipment during for the accurate measurement of maximum daily air temperature, 2) lack of simultaneous recording pre-2007 to quantify the differing sensitivities of recording equipment and 3) missing data. We describe here how we addressed these challenges.

Maximum daily temperature is usually the highest temperature recorded during daylight hours and is strongly influenced by surface solar radiation, while minimum daily temperature usually occurs at night and is therefore unaffected by irradiance (Bristow & Campbell 1984; Dai et al. 1999). While various attempts were made to shade the recording equipment at Lopé, it has since been shown that specialised solar radiation shields are necessary for accurate recording of maximum daily air temperature (Jenkins 2014; Bell et al. 2015; da Cunha 2015). However recent experience at Lopé using specialist TinyTag solar shields has shown that they increase the likelihood of termite invasion, which has resulted in equipment failure in some cases. The relative exposure of the savanna site (away from the forest canopy) and the

dynamic nature of the shaded forest site (increasing and decreasing irradiance in response to canopy changes) has interacted with the differing sensitivities of each recording unit and led to variability in the means and ranges of maximum temperature data derived from different equipment used at Lopé (Figure 2.2).

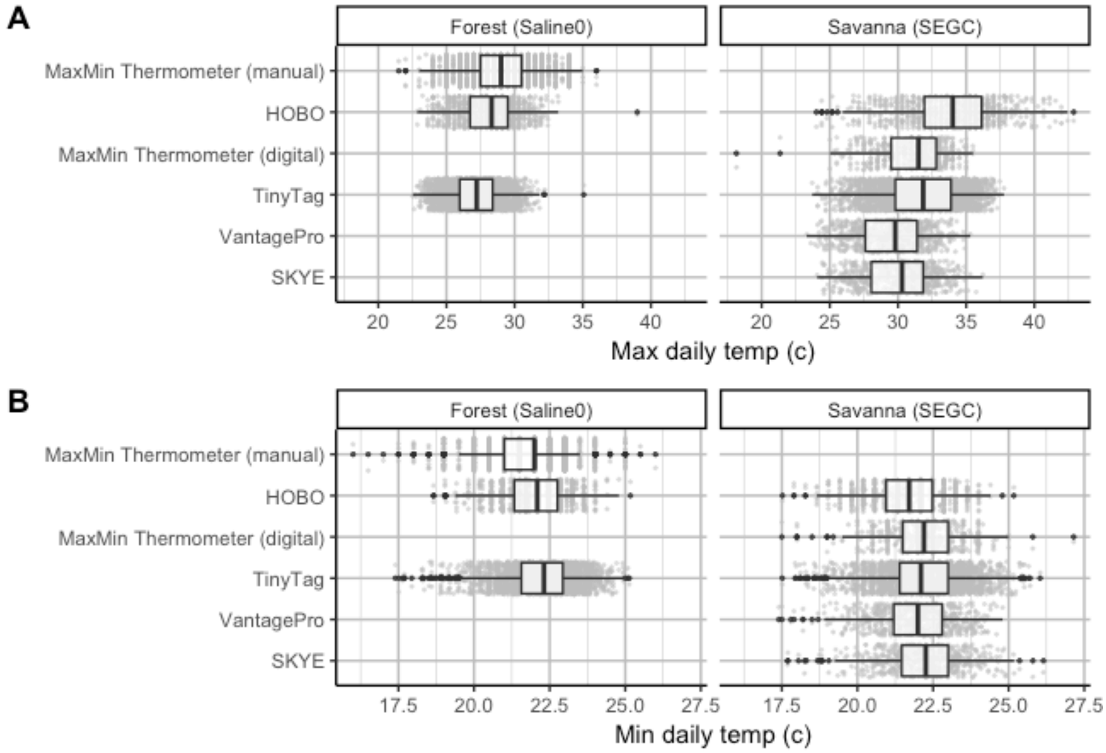

Figure 2.2. Boxplots of maximum (A) and minimum (B) daily temperature at Lopé NP, 1984-2018. Lopé forest observations cover the period 1984 - 2018 and Lopé savanna observations cover the period 2002 – 2018. Grey dots show the daily data collected at both sites (forest and savanna) using different equipment. Boxplots show the median (vertical bar), interquartile range (25th and 75th centiles; filled box), the normal range (no more than 1.5 times the interquartile range from the 25th and 75th centiles, horizontal black lines) and the outliers (outside of the normal range, black dots).

Because of the uncertainty associated with the maximum daily temperature data over time we chose to use both maximum and minimum daily temperature observations to assess seasonality ("day of year" and monthly means) and periodicity (Fourier and Wavelet analyses), but only minimum daily temperature for long-term assessments of change and inter-annual variability. Fourier analyses require continuous time series and so we summarised all max and min daily data (25538 daily max and min observations) to monthly means for each site. We excluded data for months with fewer than five observations and filled them using the mean value for the corresponding calendar month for the entire time series (temperature monthly dataset E; Figure 2.3).

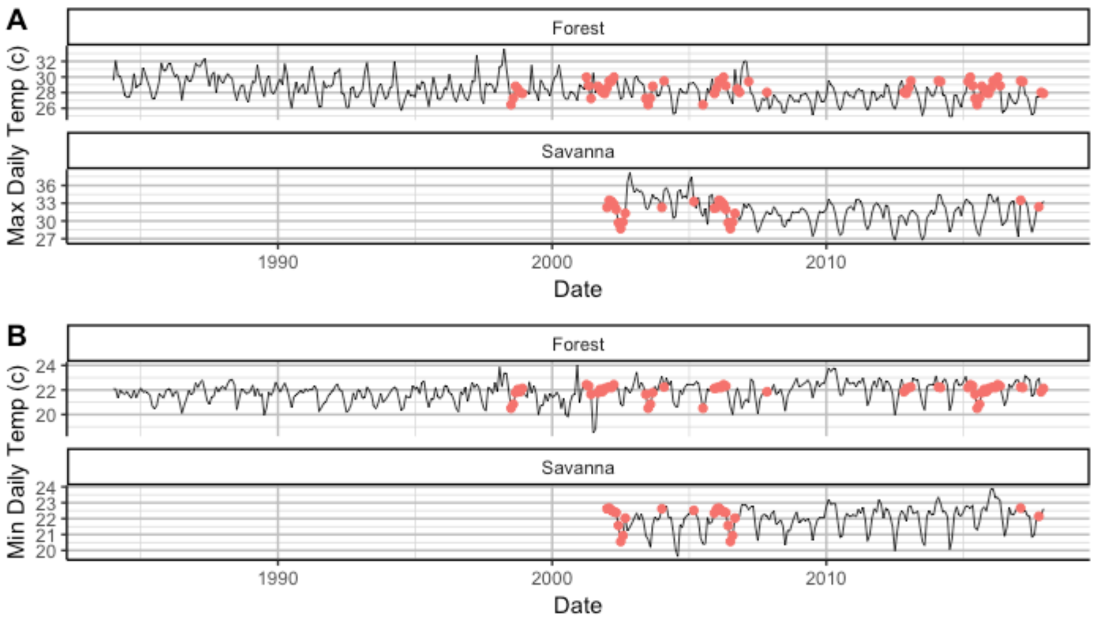

Figure 2.3. Time series plot of monthly mean maximum (A) and minimum (B) daily temperature observations at Lopé NP, 1984-2018 (Dataset E). The line shows the monthly means for each site. Red dots indicate missing months, which were filled using the mean value for the corresponding calendar month.

For long-term analyses we combined minimum temperature data from both sites (mean minimum temperature from simultaneous TinyTag recordings from the forest and savanna are 22.3°c +1.1 sd and 22.0°c + 1.2 sd respectively) and calculated the mean daily minimum temperature using all equipment for each day in the time series (8217 observations from 3/1/1984 to 31/12/2017, 34% days missing; Temperature daily dataset F). We used this daily time series for subsequent trend analyses. For analyses of the influence of ocean temperatures on temperature at

Lopé we calculated the average daily low temperature for each month excluding months with fewer than five observations (overall mean number of observations per month for all equipment = 22.3; Temperature monthly dataset G). In this monthly minimum temperature record there were 36 monthly observations missing (9% total number of months) between January 1984 and December 2017.

All data selection and manipulation procedures described can be followed in the accompanying flow chart (Figure 2.4) and R code (Code S1).

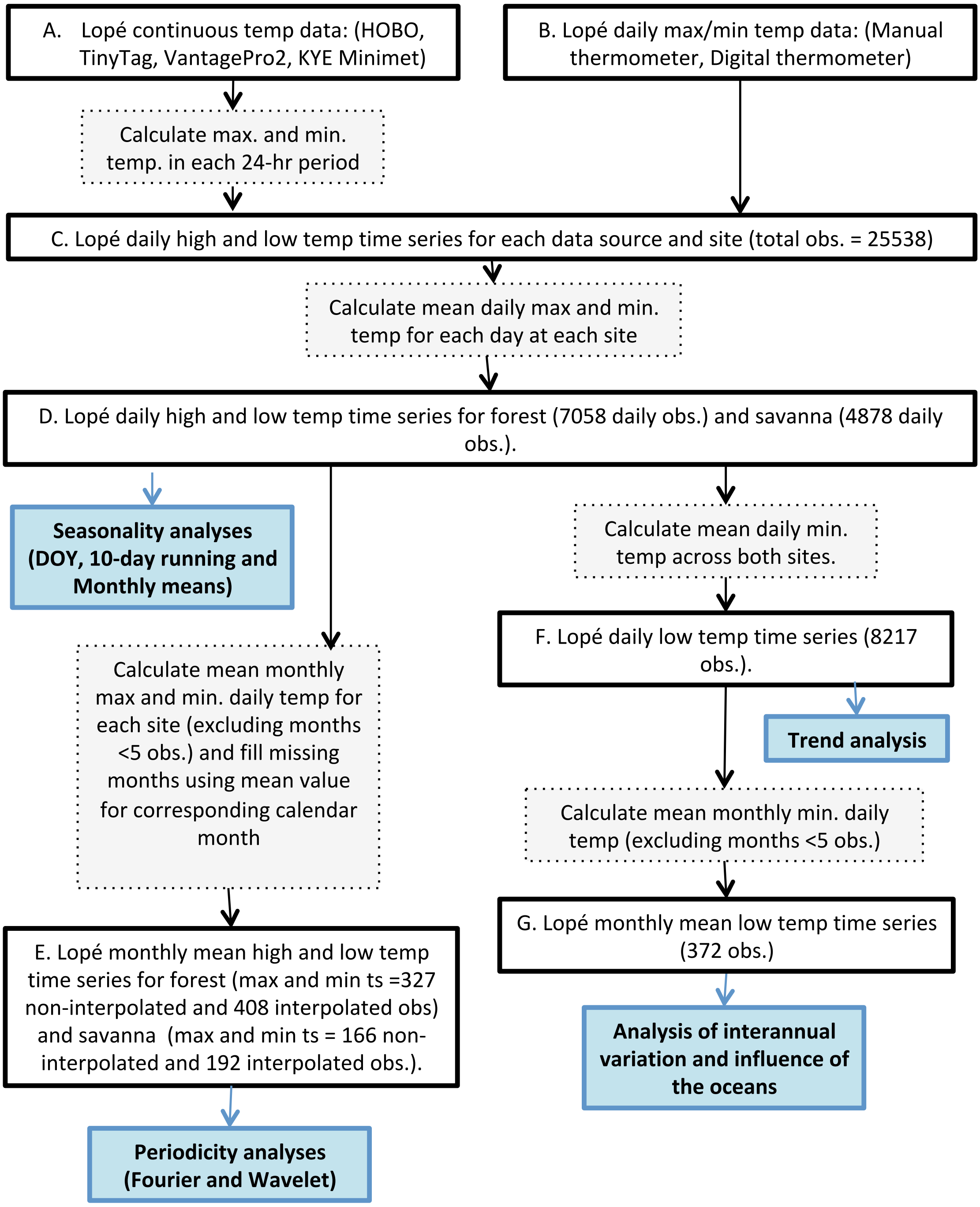

Figure 2.4. Flow chart to show data selection and manipulation steps for Lopé temperature analyses. Bold boxes indicate derived datasets, grey dashed boxes indicate processes and blue boxes indicate subsequent analyses.

3. Summary of humidity data recorded at Lopé NP, Gabon (1984 – 2018) and derived datasets

3.1 Sources of humidity data

Relative humidity has been recorded at Lopé using five different types of equipment (wet/dry bulb, HOBO units, TinyTags, and both a SKYE and a VantagePro weather station) at two sites (a savanna site: 11.605E, -0.201N and a forest site: 11.605E, -0.206N) from 1984 to the present.

From 1984, relative humidity data were recorded using a wet/dry bulb located at the forest site (1.5m aboveground under closed canopy), which was checked whenever field teams passed it or daily when logistics permitted. In 2002 all humidity recording at the forest site was transferred to continuous automatic units (ONSET HOBO® Data Loggers refhttps://www.onsetcomp.com/). Humidity recording using the same units also began in the savanna. Due to technical failures these units were removed in 2006. Other automated units (TinyTag Plus 2, Gemini Data Loggers https://www.geminidataloggers.com/data-loggers/tinytag-plus-2, some of which recorded relative humidity) were deployed in the forest from 2007 and in the savanna from 2008 and used until the present (with a gap at the forest site from mid-2015 to mid-2016 and intermittent recording throughout 2017 partly due to termite infestation). Two weather stations were installed in the savanna (sited near the research station, on a rock 4m from the ground) between 2012 and 2016. A Davis VantagePro2 (https://www.davisinstruments.com/solution/vantage-pro2/) was installed in January 2012 and recorded relative humidity every 30 minutes for two years until the equipment was struck by lightning in January 2014. A SKYE MINIMET weather station (https://www.skyeinstruments.com/minimet-automatic-weather-station/) was installed at the same location in 2013 and collected relative humidity data every 30 minutes. The SKYE unit ran intermittently until 2016 when the equipment was also damaged by lightning: data records between January 2014 and November 2014 were also lost.

To avoid errors in our dataset associated with direct solar radiation we separated day (6am-6pm) and night (6pm-6am) observations for data derived from the automatic units and calculated the mean humidity for each session per 24-hour period (Figure 3.1).

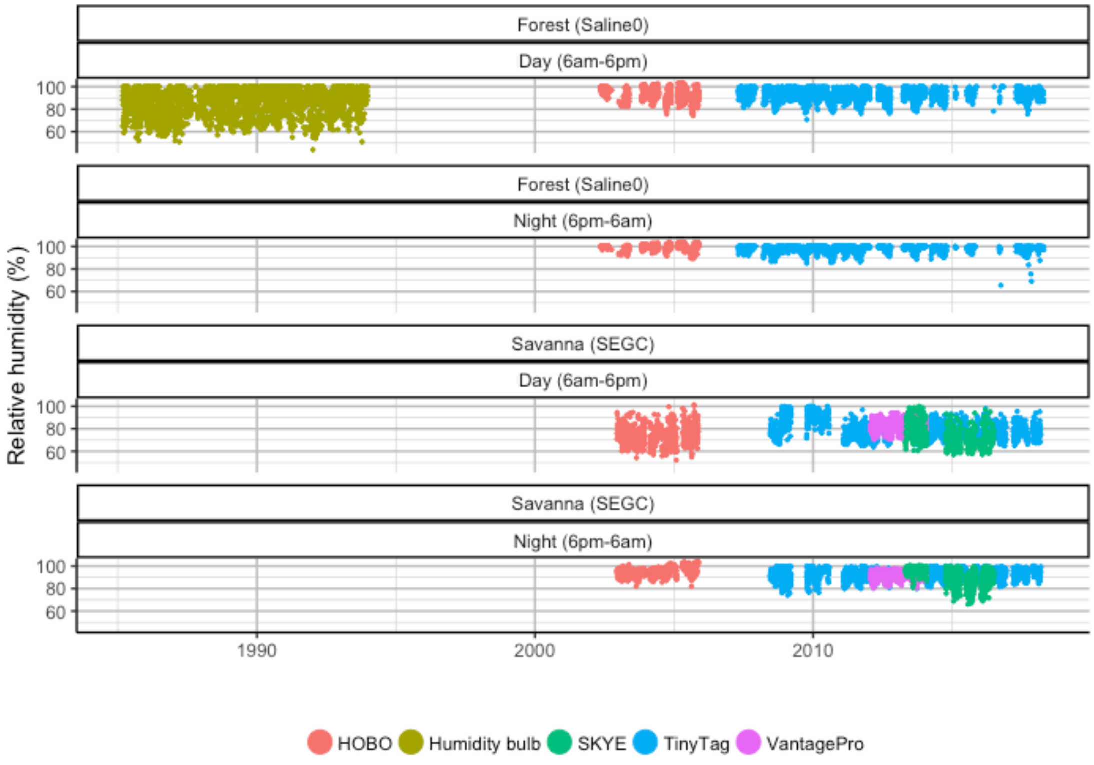

Figure 3.1 Time series plots of day and night relative humidity at Lopé NP 1984-2018. Coloured dots show daily observations from both sites (forest and savanna) and different equipment. Humidity bulb observations were recorded once per day at different times. The remaining observations are mean values for the day (6am – 6pm) or night (6pm – 6am) from automated equipment (HOBO, TinyTags, SKYE and VantagePro weather stations) with observation intervals up to 30 minutes long.

In order to reduce the impact of temperature on humidity we converted relative humidity (%) to absolute humidity (g/m^3) using simultaneous temperature records within the R package “humidity” (Cai 2018). The data available to download from the University of Stirling’s DataSTORRE (http://hdl.handle.net/11667/133) includes daily mean absolute humidity from each automatic recording unit across both sites (Humidity daily dataset A).

3.2. Addressing major challenges and data selection

We encountered four major challenges in creating a Lopé humidity record for long-term analyses: 1) the erroneous impacts of direct solar radiation and water saturation on humidity measurements and the differing sensitivities of recording equipment to these disturbances, 2) inconsistent observation times pre-2002 using the wet-dry bulb, 3) drift in measurement errors over time combined with low frequency of equipment calibration and 4) missing data.

The TinyTag manufacturers (GEMINI) advised us that erroneous relative humidity readings of 0% represented a shorting of the internal circuit under conditions of water saturation and so we removed all zeroes before from the data before further analysis. In 2016/17 all TinyTag humidity units were calibrated in the UK and were found to be over measuring relative humidity by 3.9 - 13.4% (at an applied humidity of 77-78%).

Following these checks, two units were replaced and the humidity channels on the remaining units were adjusted. However it is very difficult to know when the measurement drift occurred between 2010 and 2016 and how to remove this error.

We know that direct solar radiation leads to erroneous underestimates of humidity (da Cunha 2015) and that accurate data requires specialised solar screens, which were not used at Lopé before 2017. To avoid errors in our dataset associated with direct solar radiation we separated day (6am-6pm) and night (6pm-6am) observations for the data derived from the automatic units and calculated the mean humidity for each session per 24-hour period (6am-6am; Figure 3.1) and restricted further analyses to night-time data only. This precluded data derived from the humidity bulb as it was always collected during the daytime (usually between 7am and 6pm). In any case, it proved difficult to use the humidity bulb data for seasonal or inter-annual analyses as the data were recorded at different times each day depending on the research station schedule.

To summarise, for assessments of seasonality (Day of Year and Monthly means) we created a daily time series of mean night-time absolute humidity for days with multiple observations (Humidity daily dataset B). For Fourier analysis, which requires a complete time series, we took the mean value for each month in the record (excluding months with fewer than five observations) and filled gaps using the mean value for the corresponding calendar month for the entire time series (Humidity monthly dataset C; Figure 3.2).

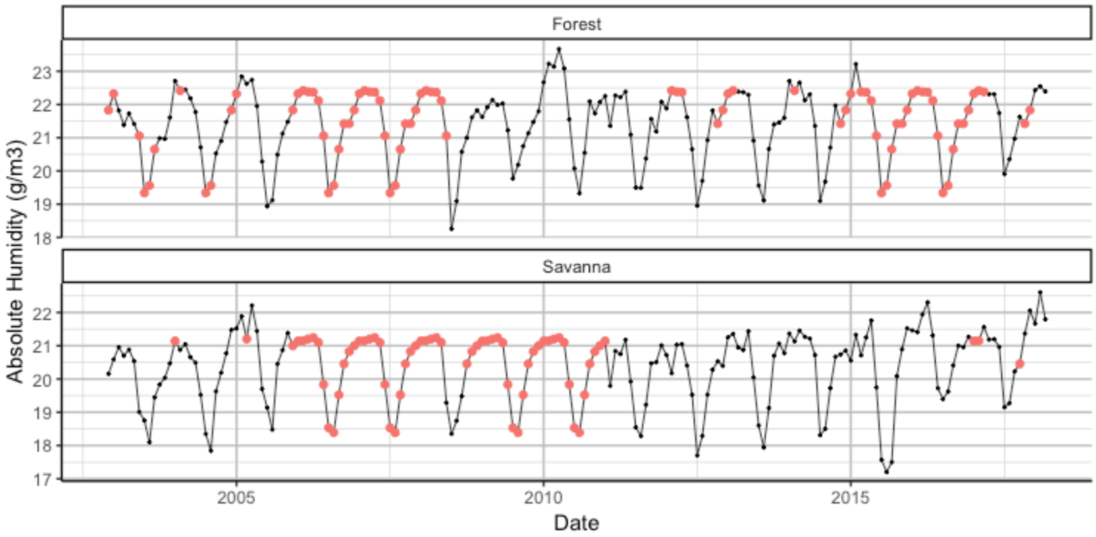

Figure 3.2. Time series plot of monthly mean relative humidity observations at Lopé NP, 2007-2018 (Dataset C). The line shows the monthly means for each site. Red dots indicate missing months, which were filled using the mean value for the corresponding calendar month.

Because of the lack of calibration between equipment and the patchiness of the data we chose not to include humidity data in further analyses of long-term change and inter-annual variability. All data selection and manipulation procedures described above can be followed in the accompanying R code (Code S1).

4. Summary of wind speed data recorded at Lopé NP, Gabon (2012 – 2016) and derived datasets

4.1 Sources of wind speed data

Wind speed has been recorded at Lopé using two weather stations - a Davis VantagePro2 (https://www.davisinstruments.com/solution/vantage-pro2/) and a SKYE MINIMET weather station (https://www.skyeinstruments.com/minimet-automatic-weather-station/) - installed at the savanna site (near the research station, on a rock 4m from the ground) between 2012 and 2016.

The VantagePro recorded data every 30 minutes for two years until the equipment was struck by lightning in January 2014. The SKYE station was installed in 2013 and collected data intermittently until 2016 when the equipment was also damaged by lightning: data records between January 2014 and November 2014 were also lost. The weather stations collected data in intervals up to 30 minutes long. We calculated the mean daily wind speed for each weather station (Figure 4.1).

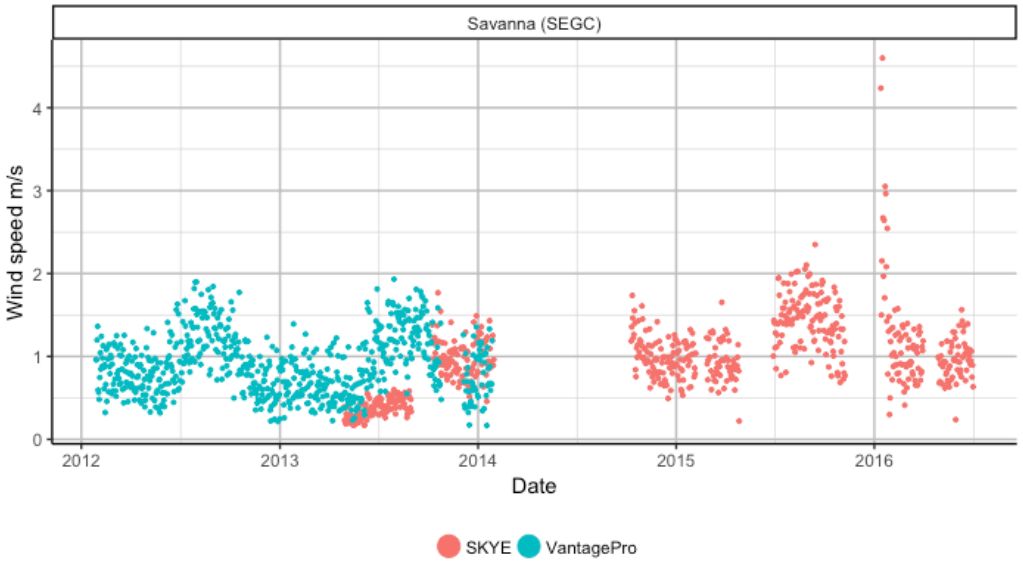

Figure 1. Time series plot of wind speed at Lopé NP, 2012-2017. Coloured dots show daily mean observations from different equipment.

The data available for download from the University of Stirling's DataSTORRE (http://hdl.handle.net/11667/133) contains mean daily wind speed data from both weather stations (wind speed daily dataset A).

4.1 Data selection

For assessments of seasonality (Day of Year and Monthly means) we created a daily time series of wind speed by taking the mean value for each day with multiple records (Wind speed daily dataset B). For Fourier analysis, which requires a complete time series, we calculated the mean value for each month in the record (excluding months with fewer than five observations) and filled gaps using the mean value for the corresponding calendar month for the entire time series (Wind speed monthly dataset C; Figure 4.2).

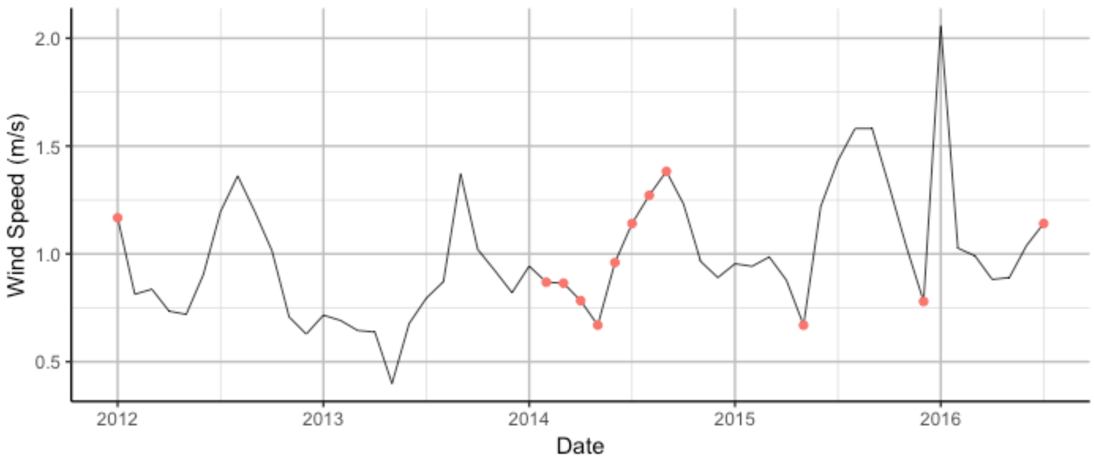

Figure 4.2. Time series plot of monthly mean wind speed observations at Lopé NP, 2012-2016. The line shows the monthly mean. Red dots indicate missing months, which were filled using the mean value for the corresponding calendar month.

Because the wind speed dataset is short and patchy we can only use it to demonstrate seasonality and periodicity not long-term trends or interannual variation. All data selection and manipulation procedures described above can be followed in the accompanying R code (Code S1).

5. Summary of solar radiation data recorded at Lopé NP, Gabon (2012 – 2016) and derived datasets

5.1 Sources of solar radiation data

Solar radiation has been recorded at Lopé using two weather stations in the savanna (11.605E, -0.201N) from 2012 to 2016. A Davis VantagePro2 (https://www.davisinstruments.com/solution/vantage-pro2/) was installed in January 2012 and recorded solar radiation every 30 minutes for two years until the equipment was struck by lightning in January 2014. A SKYE MINIMET weather station (https://www.skyeinstruments.com/minimet-automatic-weather-station/) was installed at the same location in 2013 and collected solar radiation data every 30 minutes. The SKYE unit ran intermittently until 2016 when the equipment was also damaged by lightning: data records between January 2014 and November 2014 were also lost.

We summarised the data by calculating the mean solar radiation per day for each weather station (Figure 5.1).

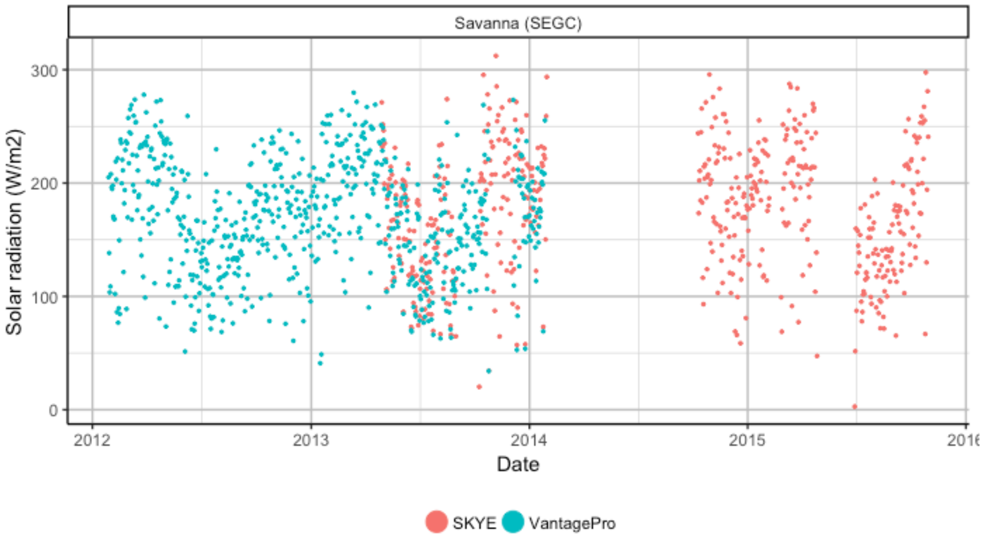

Figure 5.1. Time series plots of surface solar radiation at Lopé NP, 2012-2016. Coloured dots show daily mean observations from different equipment.

The data available to download from the University of Stirling's DataSTORRE (http://hdl.handle.net/11667/133) includes daily mean solar radiation for each weather station (solar radiation daily dataset A).

5.2 Data selection

For assessments of seasonality (Day of Year and Monthly means) we created a daily time series of solar radiation by taking the mean value for each day with multiple records (solar daily dataset B). For Fourier analysis, which requires a complete time series, we took the mean value for each month in the record (excluding months with fewer than five observations) and filled gaps using the mean value for the corresponding calendar month for the entire time series (solar monthly dataset C; Figure 5.2).

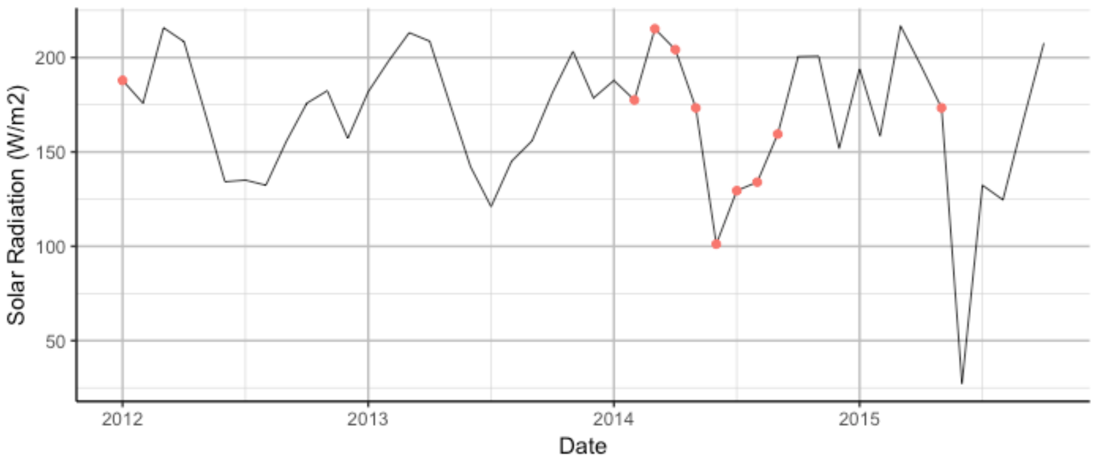

Figure 5.2. Time series plot of monthly mean solar radiation observations at Lopé NP, 2012-2016 (Solar radiation monthly dataset C). The line shows the monthly means for each site. Red dots indicate missing months, which were filled using the mean value for the corresponding calendar month.

Because the dataset is short and patchy we chose not to use it to assess long-term trends or interannual variation. All data selection and manipulation procedures described above can be followed in the accompanying R code (Code S1).

6. Summary of aerosol optical thickness data recorded at Lopé NP, Gabon (2014– 2018) and derived datasets

6.1 Source of aerosol optical thickness data

We downloaded the AERONET level 2.0 data product for Lopé (automatically cloud-cleared and manually quality assured) from the NASA Aerosol Robotic Network (Aeronet; https://aeronet.gsfc.nasa.gov/; Holben et al. 1998; AOT daily dataset A) and extracted data for the wavelengths 440, 550 and 675nm as relevant for photosynthetically active radiation (AOT daily dataset B; Figure 6.1;).

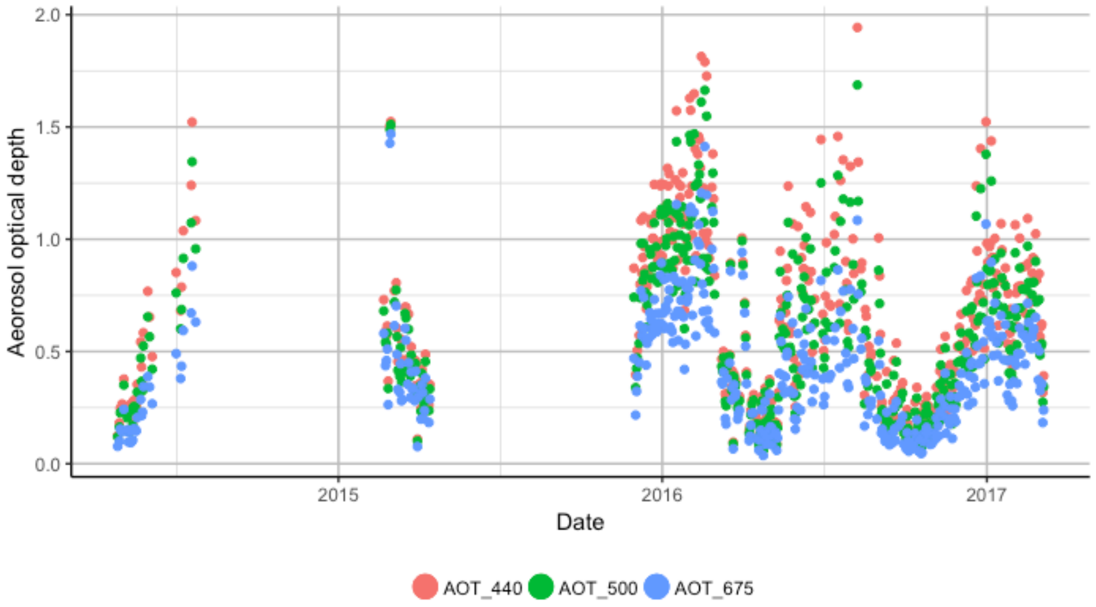

Figure 6.1. Time series plot of Aerosol Optical Depth at Lopé NP, 2014-2018 (AOT daily dataset B). Coloured dots show daily mean observations at different aerosol optical depths relevant for photosynthetically active radiation (440, 500 and 675nm).

Due to equipment error and data removal due to cloudiness, 61% of data between 24 04-2014 and 06-03-2017 is missing. Data availability is strongly seasonal and is most sparse in the months June-November (85% data points are missing in August) and most dense in March (only 35% data points missing).

6.2 Data selection

For assessments of seasonality (Day of Year and Monthly means) we used the daily time series of aerosol optical thickness for relevant wavelengths (AOT daily dataset B). For Fourier analysis, which requires a complete time series, we took the mean value for each month in the record (excluding months with fewer than five observations) and filled gaps using the mean value for the corresponding calendar month for the entire time series (AOT monthly dataset C; Figure 6.2).

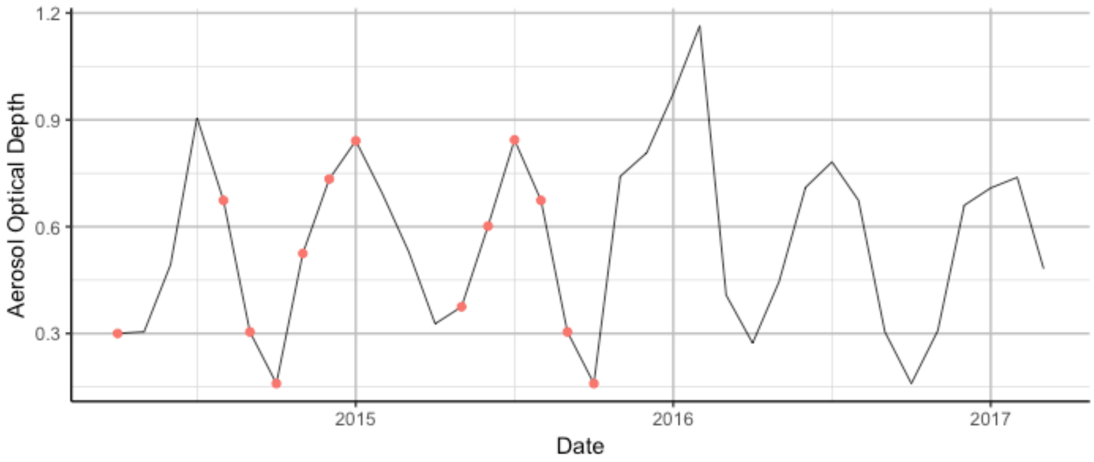

Figure 6. 2. Time series plot of monthly mean Aerosol Optical Depth observations at Lopé NP, 2012-2017 (AOT monthly dataset C). The line shows the monthly mean. Red dots indicate missing months, which were filled using the mean value for the corresponding calendar month.

Because the dataset is short and patchy we can only use it to demonstrate seasonality and periodicity not long-term trends or interannual variation. All data selection and manipulation procedures described above can be followed in the accompanying R code (Code S1).
